# *In silico* identification of conserved miRNAs and their selective target gene prediction in indicine (*Bos indicus*) cattle

**DOI:** 10.1371/journal.pone.0206154

**Published:** 2018-10-26

**Authors:** Quratulain Hanif, Muhammad Farooq, Imran Amin, Shahid Mansoor, Yi Zhang, Qaiser Mahmood Khan

**Affiliations:** 1 Key Laboratory of Animal Genetics and Breeding and Reproduction of Ministry of Agriculture, National Engineering Laboratory for Animal Breeding, College of Animal Science and Technology, China Agricultural University, Beijing, China; 2 Bioinformatics and Computational Biology Laboratory, National Institute for Biotechnology and Genetic Engineering, (NIBGE), Faisalabad, Pakistan; 3 Pakistan Institute of Engineering and Applied Sciences, Islamabad, PK; 4 Environmental Toxicology Laboratory, National Institute for Biotechnology and Genetic Engineering, (NIBGE), Faisalabad, Pakistan; University of Illinois, UNITED STATES

## Abstract

The modern cattle was domesticated from aurochs, sharing its physiological traits into two subspecies *Bos taurus* and *Bos indicus*. MicroRNAs (miRNAs) are a class of non-coding short RNAs of ~22nt which have a key role in the regulation of many cellular and physiological processes in the animal. The current study was aimed to predict and annotate the potential mutations in indicine miRNAs throughout the genome using *de novo* and homology-based *in silico* approaches. Genome-wide mapping was performed in available indicine assembly by the homology-based approach and 768 miRNAs were recovered out of 808 reported taurine miRNAs belonging to 521 unique mature miRNA families. While 42 precursors were dropped due to lack of secondary miRNA structure, increasing stringency or decreasing similarity between the two genomes’ miRNA. Increasing tendency of miRNAs incidence was observed on chr5, chr7, chr8, chr12 and chr21 with 19 polycistronic miRNA within 1-kilobase distance throughout the indicine genome. Notably, 12 miRNAs showed copy number variation. Eighteen miRNAs showed a mutation in their mature sequences in which eight were found in their seed region. Whilst in *de novo* based approach, 12 novel potential miRNAs on Y chromosome in indicine cattle along with a new miRNA (bind-miR-1264) on chrX were found. The final data set is annotated and explains the impending target genes that are responsible for enhanced immunity, heat tolerance and disease tolerance regulation in indicine. The study conforms to better understanding and perceptive approach towards indicine genome.

## Introduction

MicroRNA (miRNAs) are a class of non-coding short RNAs [1, 2] that regulate the gene expression by post-transcriptional gene silencing by catalyzing the cleavage or by translational repression of target messenger RNA (mRNA) [3, 4]. They play important regulatory roles in both animals [5, 6] and plants [4, 7] as well as found in viruses [8]. Many cellular and physiological processes are regulated by them. Predominantly, miRNA in animals can bind to 3’ untranslated regions (3’ UTR) [9–11], coding regions and to the 5’ end untranslated region (5’ UTR) [12, 13] with non-perfect complementarity. Whereas, in plants, the more dominant behavior is cleavage of the target mRNA by perfect complementarity within the target mRNA [14, 15]. The biogenesis of miRNA starts from initial transcription of long primary transcripts leading towards RNA silencing complex (RISC) assembly. The recognition of the target miRNA mainly relies on the complementarity of the seed region within the target mRNA [12, 13].

The differential expression of miRNA [16, 17] and its polymorphism [18, 19] has been concomitant with disease, immunity and various phenotypic traits. The seed region around 2 to 9 nucleotides from the 5’-end of miRNA is the key binding region for miRNA target recognition [20, 21]. The stability of precursor miRNA, as well as target specificity of mature miRNA, rely on mutations in the mature sequences, especially in the seed region [9].

Livestock production has been the heart of the socio-economic system in the tropical and non-tropical regions of the world. The domesticated cattle, originating from aurochs, is subdivided into two closely related sub-species mainly, *Bos taurus* (taurine) and *Bos indicus* (indicine or zebu). Taurine is also known as European cattle and has been extensively studied due to its early puberty, high fertility and increased productivity in terms of milk and meat [22–24]. On the other hand, indicine is a tropical cattle which confers extraordinary physiological traits including enhanced heat tolerance, stronger immunity and resistant to various diseases due to its superior ability of thermo-tolerance and regulation of body temperature along with its intrinsic cellular resistance [22, 25]. As per studies, over 50% of the bovine population is located in the tropics and heat stress causes approximately over 60% economic loss in dairy farms because augmented milk enhances the susceptibility towards deleterious effects of heat stress [19, 22, 26]. Both of the species play an intrinsic role in meeting the dairy and beef consumption. However, indicine genome can be further reconnoitered for its better survival ability in the least veterinary care and endurance in the tough atmosphere [19, 26, 27].

The complete taurine genome has been published and various assembly versions *e*.*g*. Btau5.0 from Baylor college of medicine (https://www.hgsc.bcm.edu/other-mammals/bovine-genome-project) and UMD 3.1.1 from University of Maryland (http://bovinegenome.org/?q=node/61) are available. Whereas, a genome of indicine breed (Nellore, Brazil) has also been published by taking Btau4.6.1 as a reference genome with high coverage and assembly statistics [28]. The previously annotated miRNAs in mirBase-v21 are deposited with reference to *Bos taurus* assembly UMD3.1.1; the variation including frame shifts, the orientation of the two genomes as well as gain or loss can play the significant role in regulating the two breeds [29]. So it is important to perform a comparative study of miRNA genes including their variations in copy numbers and mature sequences to elucidate complete miRNA genes’ annotation between these two assemblies. This can lead to a repository of useful ncRNA knowledge for further gene regulation studies of tropical breeds, taking indicine (Nellore, Brazil) as a reference.

At present, 2588 miRNAs from human and 1915 of mouse genome have been deposited in miRBasev21 [30]. There are still clusters of various miRNA families found in humans or other related species that are absent or yet to be discovered in bovine. The initial studies on miRNA expression in taurine tissues resulted in 793 (incl. copy number variant; CNVs) miRNAs, encoded on 30 chromosomes, all aligned with reference to *Bos taurus* assembly UMD 3.1 [31]. The release of indicine assembly covering 97% of the taurine genome in 2.5Gb provides a unique opportunity for a deeper and more comprehensive characterization of tropical cattle’s miRNA transcriptome using computational methods [28].

In the present study, we used both *de novo* and homology-based approaches to predict novel as well as taurine miRNA sequences in indicine genome. This data will create a baseline miRNA information for the reference indicine genome. The final data set is annotated, along with polymorphism in mature miRNA transcripts, target prediction and comparison between both cattle species and copy number variations.

## Methods

### Data sources

MiRBase is the main data repository for the storage of miRNA information from different experimental and computational discoveries from various species [30]. Reference taurine miRNAs along with 18687 mature miRNAs from 51 species from chordate’s family were taken from miRBase 21 (http://www.mirbase.org/) for the analysis. The reference indicine cattle genome assembly was taken from National Center for Biotechnology Information, accession: GCA_000247795.2 [28].

### Bioinformatics data mining

#### Search for taurine miRNAs in indicine genome

Genome-wide miRNA homology-based searches were made using Bowtie2 [32] and NCBI blast+ (Blastn algorithm) tool using taurine miRNA precursor sequences against the assembled genome of indicine. The mapped sequences were inspected visually employing Tablet genome viewer [33]. The resultant sequences were checked for their secondary structures using MFold [34, 35], on the basis of the minimum free energy index (MFEI≤-0.85) [36] calculated according to the percentage and ratio of A+T and G+C content [5, 37]. The value of MFEI was selected as ≤-0.85 and further downstream analysis was performed using shell and Perl scripts under Linux environment [38]. The mature sequences of taurine were also aligned with the indicine assembly, keeping the word size 4, with zero nucleotide mismatches allowed [39–41].

Redundant and partially overlapped sequences to the exons were filtered at each step. 150nt flanking regions were selected and excised from the genome considering their 5’ and 3’ possible potential mature sequences. The candidate precursors were then analyzed, predicted and annotated using bioinformatics tools. The complete scheme followed is tailored in a flowchart in S1 Fig.

Additionally, genome-wide new miRNAs were also predicted by mapping mature miRNA sequences of chordates, extracted from miRBase-v21 to the genome assembly of indicine, using Bowtie2. To identify the novel miRNAs in indicine genome, 18687 mature miRNA sequences from 51 families of genus chordates were selected and mapped to the indicine genome. MiREAP was employed for extraction of potential new miRNA precursors [42–44]. Furthermore, the conserved new miRNA was subjected to phylogenetic analysis by MEGA7.0 [45].

#### Identification of novel miRNA of *Bos indicus* in Y chromosome

The *de novo* prediction was executed on indicine genome, using miRPara [46, 47]. The predicted sequences were subjected to data mining, exonic mappings and redundant precursors were filtered out. The SVM probability was set to 0.99, in order to increase the stringency, MFEI ≤ -0.85 and secondary structure were confirmed using MFold. Furthermore, the precursor length, genomic coordinates, and genome locus were sternly observed for the presence of miRNA [14, 48–50]. The scheme adopted for the prediction of miRNAs is listed in S2 Fig. The predicted novel miRNAs were then subjected to phylogenetic analysis with homologous known miRNAs. The tree was statistically validated by bootstrap with 1000 replicates to ensure the accuracy. The inference was drawn between conserved and diverged miRNAs [51].

### Annotation, copy number variations (CNV) and frequency Distribution

The annotation is provided in UCSC bed file format in S1 Table. The positional clusters were created using a cluster tool from Bedtools using strand-specific (-s) and by varying the window size (-d) from 1 kb to 10kb [52, 53]. The list of all annotated miRNAs was compared in indicine and taurine to elucidate mature miRNA polymorphisms, copy number variations (CNVs), frequency distribution and possible effects of mature sequence polymorphism on precursor stability and target specificity. The candidate polycistronic clusters were further verified for the promoter prognosis, using the tool Neural Network Promoter Prediction of Berkeley Drosophila Genome Project and keeping the threshold to promoter score of 0.80 [20, 54].

### MiRNA target prediction

The tools employed for miRNA target prediction were miRNA targets [55], miRmap [56, 57] and Visualization Applet for RNA (VARNA) [58]. The alterations in the target genes were observed using the custom version of Target Scan [59, 60].

## Results

### Conserved *Bos indicus* miRNAs

#### *In silico* identification of *Bos indicus* miRNAs

Aligning the taurine miRNAs with the indicine genome along with their secondary structure confirmation allowed us to identify the conserved miRNAs in indicine cattle. Taurine and indicine are closely related species but genomic locations and origins of miRNAs differ due to different genome assembly versions [28]. Out of 808 already reported taurine miRNAs precursors, 749 (92.69%) precursors mapped perfectly with100% query coverage; 17 (0.02%) aligned within 80–99 query coverage. Only 2 (0.0026%) precursors were mapped within 79–70% query coverage and the rest of the precursor alignments below 70% were disqualified 42 (~0.050%) due to their decreasing similarity levels, lack of secondary structure formation and the increasing stringency. Overall, 736 conserved miRNAs were retrieved from the taurine genome and 12 miRNAs showed copy number variations. Out of 154 non-perfect similar precursors, 18 showed mutations in their mature sequences whereas 8 of them showed a mutation in their seed region, which were further subjected to target gene prediction [9, 61]. The distribution of miRNAs in indicine is shown in Table 1.

**Table 1:** Distribution of *B*. *indicus* miRNA sequences found, using genome mining. Out of 808 precursor miRNAs, 766 were found in the indicine genome, whereas 18 showed putative mutations in their mature sequences, 8 of them in the seed region, whereas 12 had different copy numbers between the two species.

*Bos indicus* miRNA	
Total conserved miRNAs found	736
Copy Number Variation	12
Polymorphism	18
• Seed Region Mutation	8
Not Found	42
Total	808 (As reported in *Bos taurus*)
Potential Novel miRNAs in ChrY	12
New miRNA predicted	01

**Fig 1:**
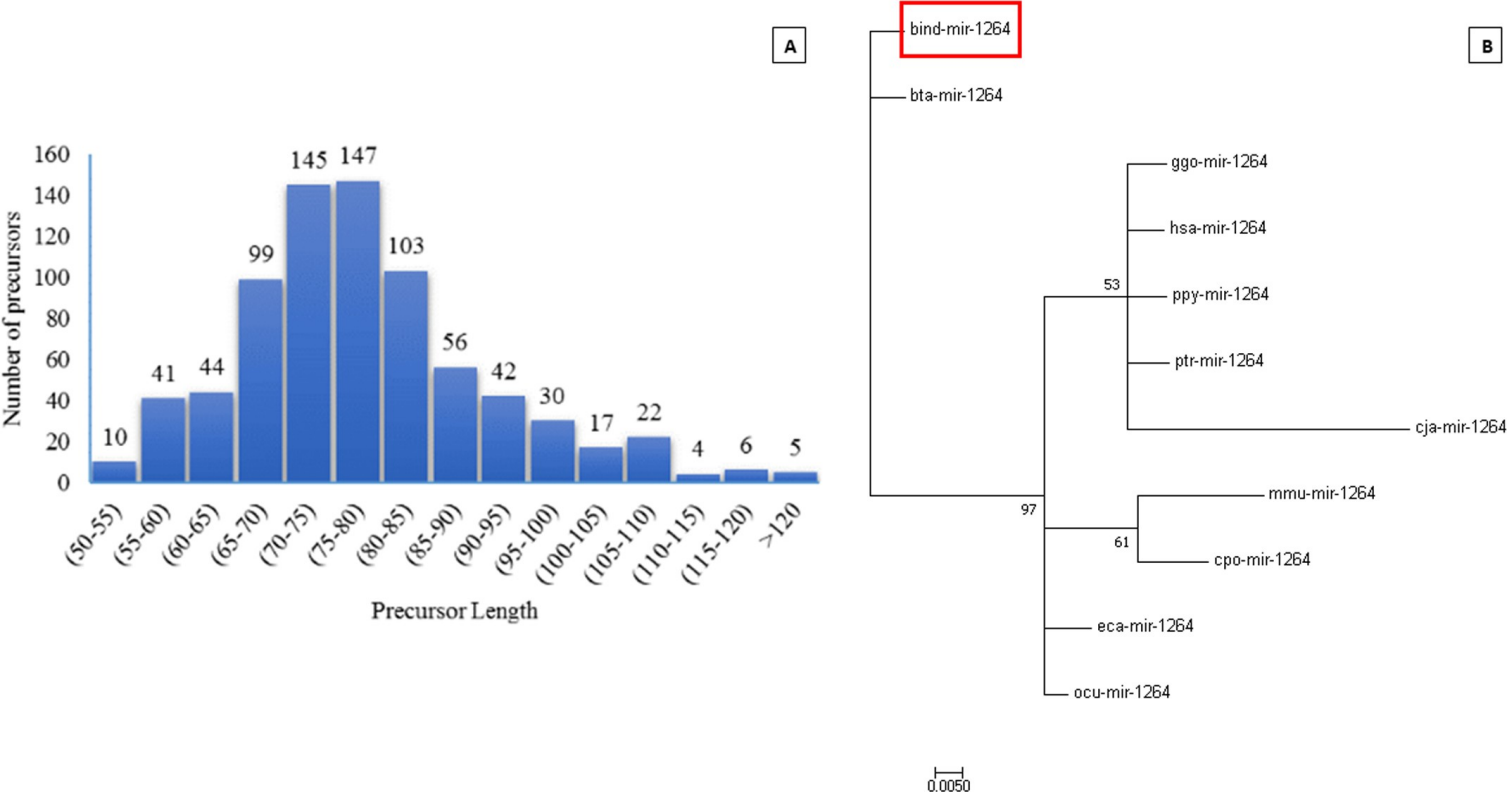
**(A) Size distribution of miRNA precursors in indicine**. Histogram shows the average size distribution of the potential pre-miRNA candidates by genome wide homology based search. **(B) The phylogenetic tree for the new miRNA** (bind-mir-1264) found under MiReap was inferred using the neighbor joining method in MEGA7. The percentage of replicate trees that are associated in clustering of taxa in the bootstrap (100) are represented next to the branches. Evolutionary analysis was conducted.

Exact duplicates and the overlapping predicted sequences were eliminated from the potential precursors. The precursor size ranged from 52 to 148nt, with the majority of the precursors lying in the range of 70 to 80nt (51.36%), 52 to 70nt (25.22%) and 85 to 110nt (21.712%). Only 3.90% shared precursor miRNA from 110 to 148nt as depicted in (Fig 1A).

The mature sequences perfectly mapped to the indicine genome, were used to excise 150nt flanking regions and tested for miRNA secondary structure criteria. Running MiReap resulted in 122 potential miRNA candidates, out of which one new miRNA (bind-mir-1264) was found in the indicine genome, fulfilling the miRNA criterion and previously reported in other closely related species Table 1 and S2 Table. The phylogenetic distance was inferred for it which showed its close relevance to the recently added taurine miRNA, validating the authenticity of the prediction criterion (Fig 1B).

### Genome-wide *de novo* prediction of novel indicine and taurine miRNAs

MiRNA prediction in indicine revealed 12 potential novel miRNAs that fulfilled all the stringent criterions [49]. These predicted potential novel miRNAs are predicted in chromosome Y and are depicted in S3 Table. The predicted novel miRNAs were also investigated for their incidence in the taurine as well as human. Taurine showed 100% homology whereas all the prophesied miRNAs were found absent in the human genome; so, they can be deliberated as a novel in the taurine genome. The phylogenetic study shows conservation of sequence signatures within the species in S1 File.

### Comparative copy number variation analysis

**Fig 2:**
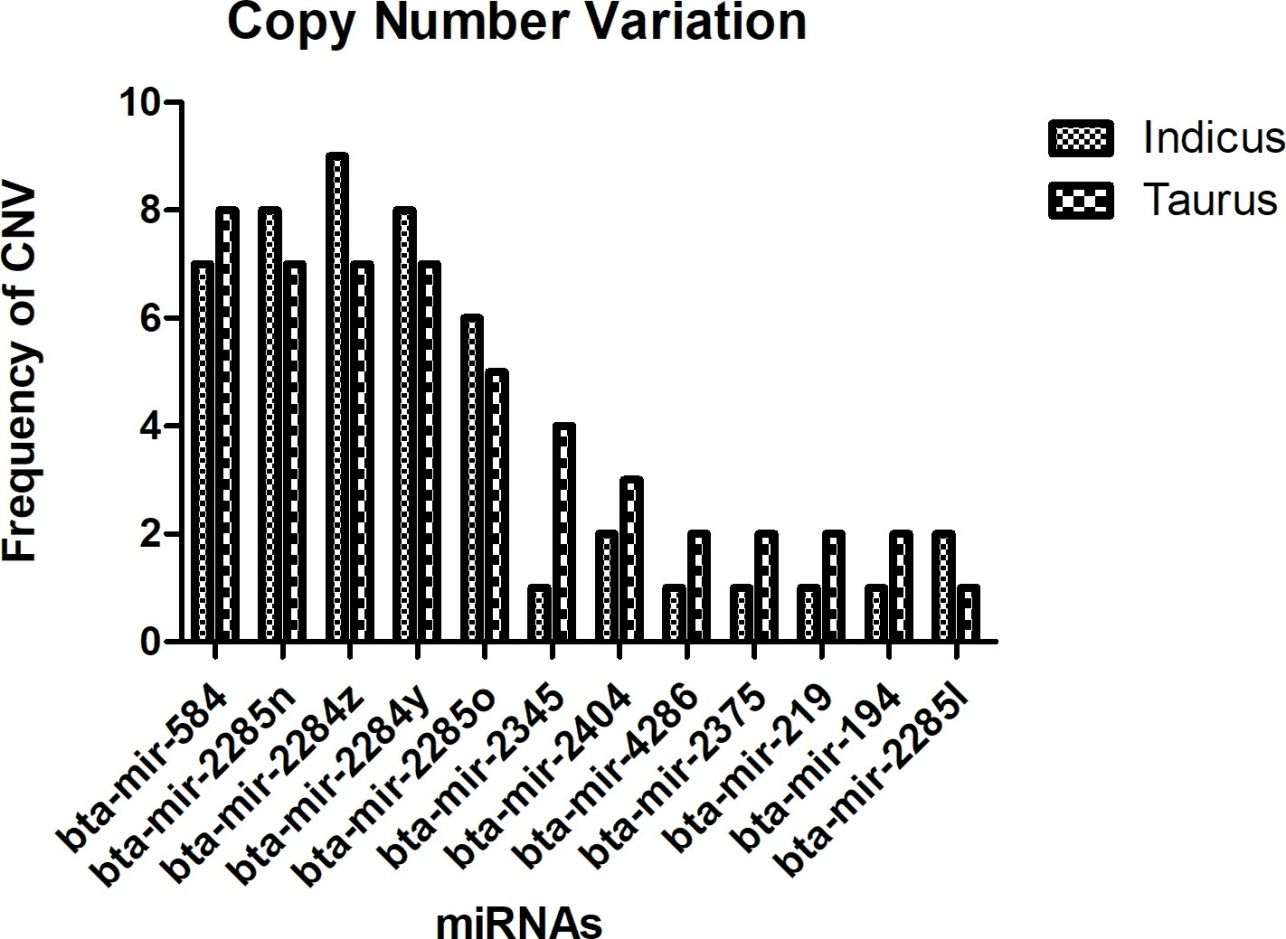
Comparative copy number variants (CNVs) in *B*. *indicus* and *B*. *taurus* in the genome. Graphs showing the fraction of miRNA loci indicates the differential expression of the some of the miRNAs in both sub-species.

The disparity in the copy numbers of miRNAs plays a significant role in the target regulation capability of animals [62]. The precursors of these copies may or may not share the same nucleotide sequences. However, the gain and loss of the miRNA genes occur from segmental duplication or tandem repeat events [63, 64]. The *in-silico* analysis revealed 12 miRNAs having variations in their gene copies, which might explain the varying adaptation and the phenotypic diversity, within these species. The highest copy number variations (CNVs) were observed in mir-2345 that has four copies in taurine and only one in indicine, whereas mir-584, mir-2404, mi-4286, mir-2375, mir-219, mir-194 showed reduced copy numbers in indicine as elaborated in (Fig 2). The mir-2284z had two extra copies in indicine, whereas mir2285n, mir-2284y, mir-2285o, and mir-2285l showed an increase in single copy numbers in indicine genome. In case of increased copy number in indicine, a new additional copy of mir-2284z was found on chromosome 15 of indicine. Similarly, mir-2285o was also found on chr5 other than the available copies in taurine. Two copies of mir-2285l were observed on chr12 and the third was present on chr2. The miRNAs, mir-584 lacked two copies on chr29 and X, mir-2285n on chr14, three copies of mir-2345 on chr19, mir-2404, mir-4286, mir-2375, mir-219 and mir-194 on chr17, chrX, chr23, chr23, and chr16 respectively. The comparative CNVs in indicine and taurine have been shown in (Fig 2).

### Genomic locations of miRNAs in *Bos indicus*

**Fig 3:**
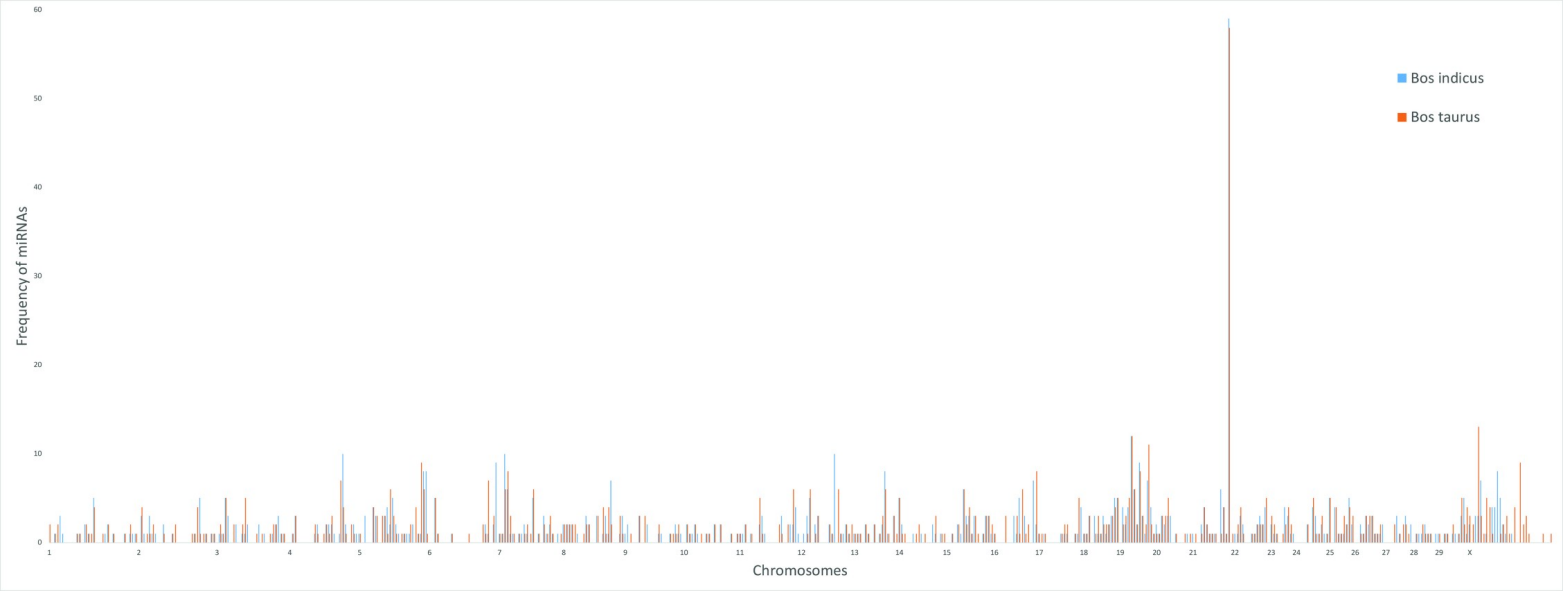
The distribution characteristics of miRNA in *B*.*indicu*s in a density plot. The location of total miRNA are shown across 31 chromosomes. The histogram bars represent the frequency of miRNA’s at a given loci. There is a significant increase in miRNA frequency at chromosome 22, X. 20, 16 by increasing order respectively (window size = 5MB).

The genomic origins of indicine (Nellore L.) identified in this study have been reported in UCSC bed file format for future references in S1 Table. Chromosome 21 has the greatest number of miRNAs (82), chromosome 19 with 55 miRNAs followed by chr5 (51), chrX (41), chr7 (39), chr8 (33), chr12 (32), chr4 (30) and as low as 8 miRNAs in Chr28 (Fig 3).

**Fig 4:**
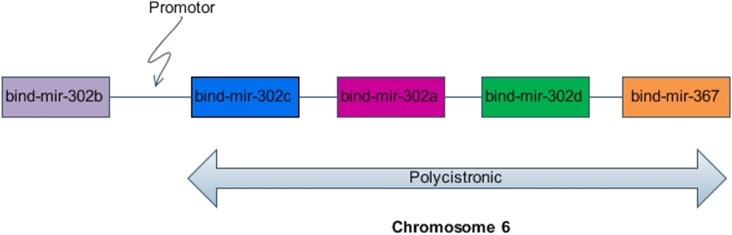
General characteristics of miRNA polycistronic clusters in B.indicus. The boxes represent polycistronic clusters of four miRNAs governed by a single promoter on chromosome 6 in *B*.*indicus*.

The miRNAs of animals tend to form more clusters than plants with varying intergenic distances [65, 66]. The nearest neighbors may form polycistronic clusters as well. A total of 24 clusters with 1kb apart were found. Detailed sequence analysis revealed that 19 of them actually had a single promoter towards 5’ end of the leftmost originated miRNA. There were no promoter sequences within the intergenic regions of a miRNA polycistronic block. Furthermore, nine polycistronic clusters were 2kb apart, one was 4kb and nine were found to be 5kb apart. The bind-mir-17, mir-106a, mir-18a, mir-18b, mir-19a, mir-20a, mir-19b, mir19b-2, mir-92a-1, mir-92a-2 shared a single cluster within 1kb window and are polycistronic. Similarly, clusters of up to 5 miRNAs within 1Kb window and clusters up to 3 miRNAs within 2Kb window size were found in S4 Table. Chromosome 4, 14, 22 and 25 showed distant clusters of 5kb apart. Increasing the window size from 1 kb to 10 kb decreases the chances of such polycistronic miRNA clusters. Fig 4 is the representation of the polycistronic clustering on chromosome 6 within 1 Kb distance (Fig 4).

### Mutations in mature miRNAs between taurine and indicine

Altogether, 766 miRNAs were annotated for the indicine genome, 18 were having mutations in their mature sequences in Table 2. Out of these 18 miRNAs, 8 showed mutations in their seed regions. The bind-mir-2284e showed seed mutation of Adenine (A) at position 7 from 5’ end instead of Guanine (G) in taurine. Similarly, bind- mir-2403 showed seed mutation at 4^th^ nucleotide in its mature sequence with Adenine instead of guanine. Bind-mir-2285l depicted up to 4 substitutional mutations in the mature sequence, where precursor homology accepted up to 4 mutations, 3 of them were found in the seed region precisely at position 4, 7, 8 and 18 from 5’ end, where Adenine (A), Cytosine (C), Guanine (G) and Thymine (T) were replaced by T, A, A and C in indicine as compared to taurine miRNA. Bind-mir-2285n-6 had 2 seed mutations of A instead of C, T instead of C at position 6^th^ and 8^th^ with a single insertion of T from 5’end. Moreover, bind-mir-2285o-5 included 3 mutations, one of them being a seed mutation at positions 2 (A into G), 10(Conversion of C into T), 19 (G converted to T). Bind-mir-6522 shared one seed mutation at position 2 (T instead of C) and one mature mutation at position 19 (converted C into T). The bind-mir-2284w shared one seed and one mature mutation at position 7 (T into A) and 12 (C into G) respectively.

**Table 2:** The Mutations found in the mature miRNA sequences are enlisted for *B*.*indicus* and *B*.*taurus* genome. The nature of mutation shows the potential change in the given miRNA.

miRNA Name	Mature miRNA name	Chromosome	Mature Taurine Seq	Mature Indicine Seq	Type of Mutation	Type	Alleles miRNA region
bta-mir-2284e	bta-miR-2284e	chrX	AAGTTC**G**TTCGGATTTTTCC	AAGTTC**A**TTCGGATTTTTCC	1S	SNP	Seed
bta-mir-2284l	bta-miR-2284l	chr3	AAAAGTT**G**GTTC**G**GGTTTTT	AAAAGTT**C**GTTC**A**GGTTTTT	2S	SNP	Mature
bta-mir-2284y-4	bta-miR-2284y-4	chr24	AAAAGTTC**G**TT**C**GGGTTTTTC	AAAAGTTC**A**TT**T**GGGTTTTTC	2S	SNP	Mature
bta-mir-2284y-7	bta-miR-2284y-7	chr3	CGGGGGTGGCGGGGAG**G**GGG	CGGGGGTGGCGGGGAG**C**GGG	1S	SNP	Mature
bta-mir-2305	bta-miR-2305	chr13	AAAAAAGTTTGT**T**TGGGTTTTTT	AAAAAAGTTTGT**G**TGGGTTTTTT	1S	SNP	Mature
bta-mir-2403	bta-miR-2403	chr28	CTC**G**GGAAGCTAGCTGGCCTT	CTC**A**GGAAGCTAGCTGGCCTT	1S	SNP	Mature, seed
bta-mir-2421	bta-miR-2421	chr4	AAAAA**C**C**C**GAATGAACTTTTTGG	AAAAA**A**C**T**GAATGAACTTTTTGG	2S	SNP	Mature, seed
bta-mir-2467	bta-miR-2467	chr8	ACCCCAAGCCTGGC**T**GCTA	ACCCCAAGCCTGGC**C**GCTA	1S	SNP	Mature, Seed
bta-mir-2284w	bta-miR-2284w	chr9	AAAAGTTC**G**TTCGGGTTTTTC	AAAAGTTC**A**TTCGGGTTTTTC	1S	SNP	Mature
bta-mir-2284z-1	bta-miR-2284z-1	chr10	CTC**G**GGAAGCTAGCTGGCCTT	CTC**A**GGAAGCTAGCTGGCCTT	1S	SNP	Seed
bta-mir-2284z-2	bta-miR-2284z-2	chr11	TATTTTTTTGTTTC**G**TGTTT	TATTTTTTTGTTTC**A**TGTTT	1S	SNP	Mature
bta-mir-2284z-5	bta-miR-2284z-5	chr5	ACCCCAAGCCTGGC**T**GCT	ACCCCAAGCCTGGC**C**GCT	1S	SNP	Mature
bta-mir-2285l	bta-miR-2285l	chr12	AAGAGT**T**TGTT**C**GGGTTT	AAGAGT**A**TGTT**G**GGGTTT	2S	SNP	Mature, seed
bta-mir-2285n-6	bta-miR-2285n-6	chr1	AAAAAAGTTT**G**TTTGGGTTTTT	AAAAAAGTTT**A**TTTGGGTTTTT	1S	SNP	Mature, seed
bta-mir-2285o-5	bta-miR-2285o-5	chr5	**A**AAAAAGTTTGTTTGGGTTTTT	**C**AAAAAGTTTGTTTGGGTTTTT	1S	SNP	Mature
bta-mir-6522	bta-miR-6522	chr21	T**C**GGAATTGTTTGTGTAC**C**TGT	T**T**GGAATTGTTTGTGTAC**T**TGT	2S	SNP	Mature, seed
bta-mir-664a	bta-miR-664a	chr28	CAGGCTAGGAGGT**GT**G**T**GTGGATG	CAGGCTAGGAGGT**AA**G**A**GTGGATG	3S	SNP	Mature
bta-mir-2285l	bta-miR-2285l	chr12	AAAACCCG**C**ATGAACTTTTT**G**GC	AAAACCCG**A**ATGAACTTTTT**A**GC	2S	SNP	Mature

**Fig 5:**
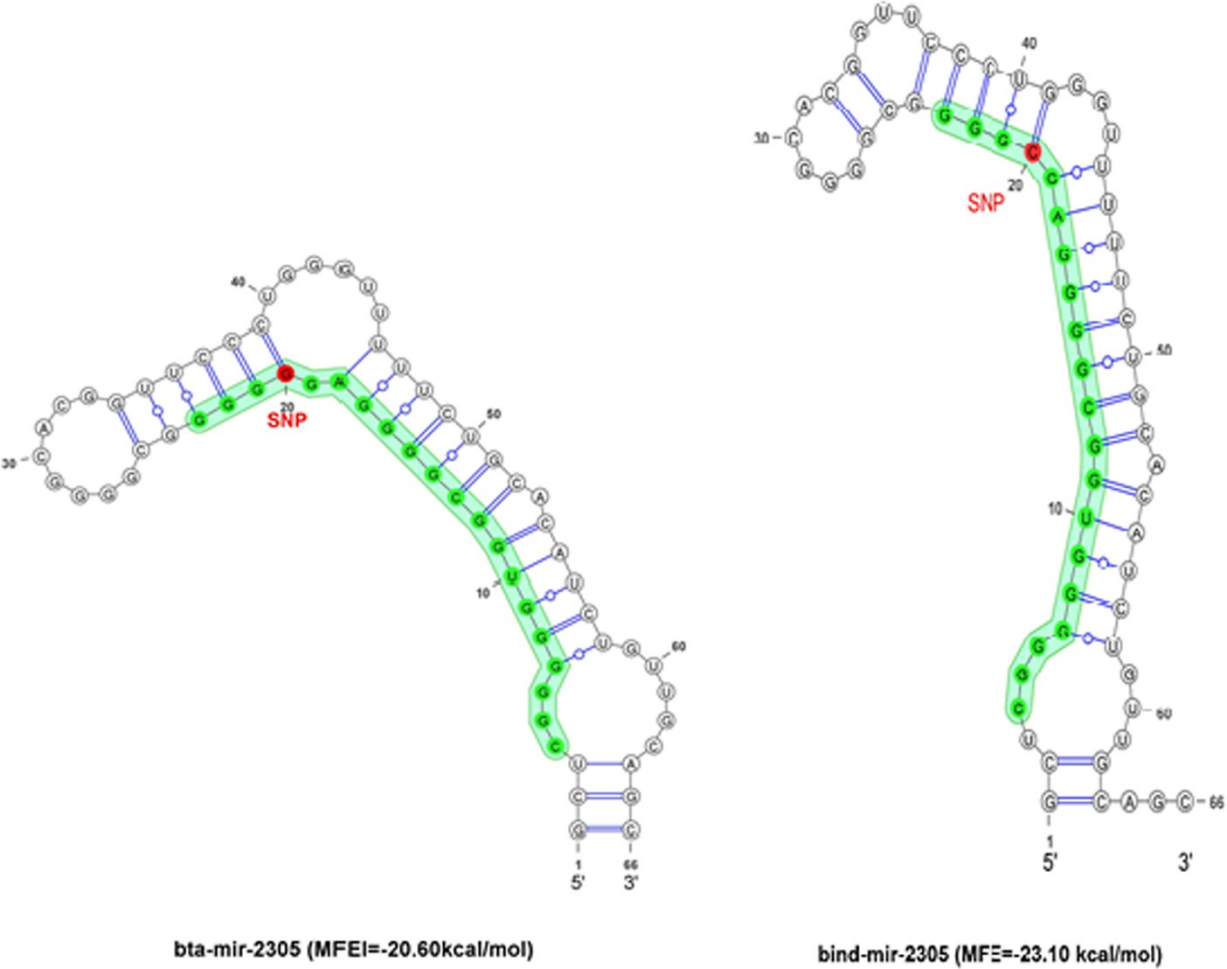
Secondary structure stability affecting miRNA regulation in *B*.*indicus* and *B*.*taurus*. The structures represent the stability and the stem loop of bind-mir-2305 and bta-mir-2305. Prediction is conducted by using MFold and by calculating its minimum free energy index (MFEI ≤-0.85).

Interestingly, the secondary structure of bta-mir-2305 and bind-mir-2305 shows the potential effect on the stability of miRNAs leading towards the activity of the miRNA where cytosine is replaced instead of guanine at position number 17^th^ and comes up with even more stable miRNA, in terms of its MFEI than in taurine cattle (Fig 5).

### Effect of seed mutations on target genes

**Fig 6:**
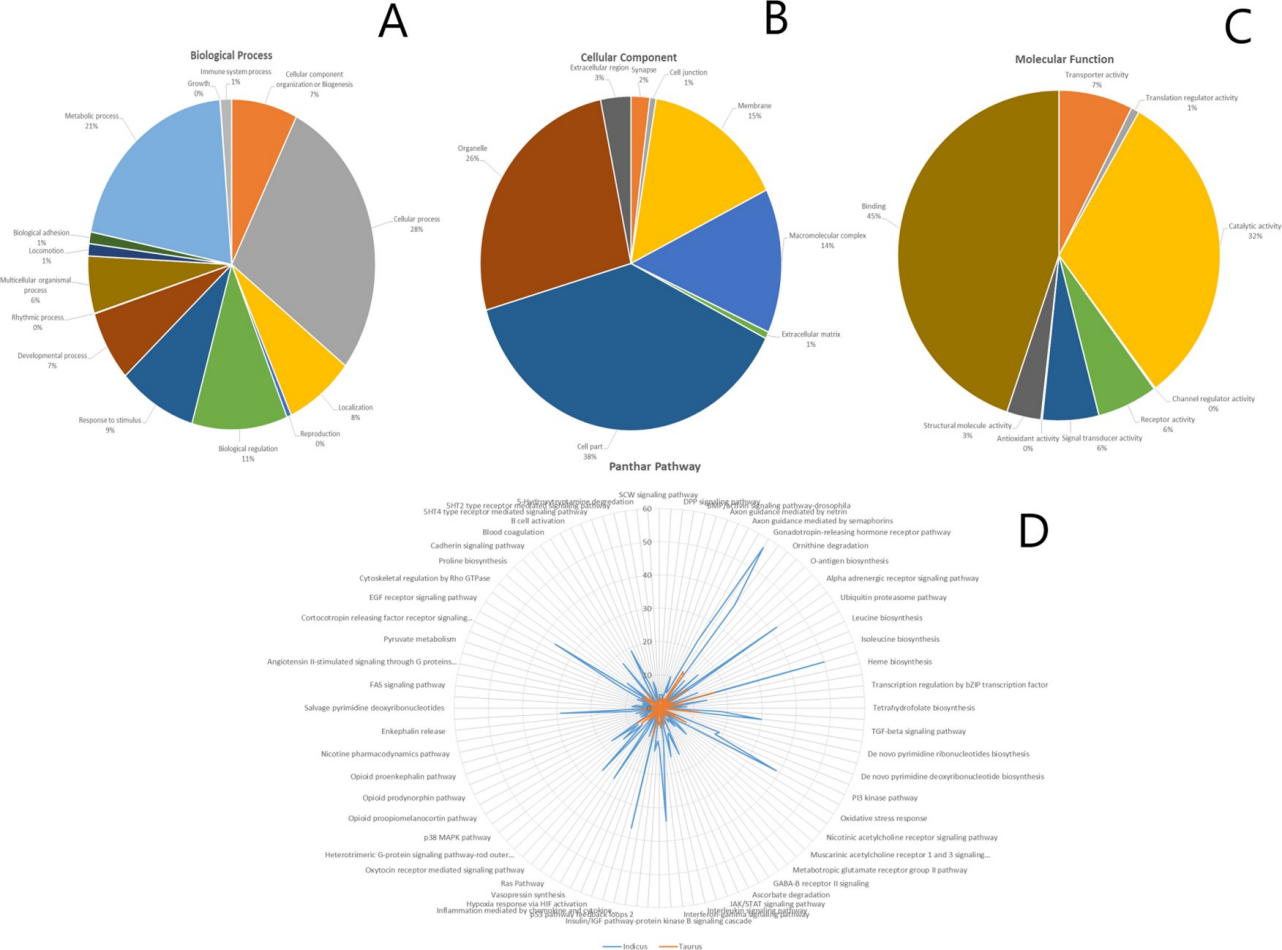
**The Panthar ontology classification based on (A)** Biological process, **(B)** Cellular component and **(C)** Molecular Functional classification of target genes in indicine. **(D)** The panther pathway depicts the significant difference in the regulation of target genes in indicine as compared to taurine.

The targets were predicted for the miRNAs which had mutations in their seed region and the possible trend towards the immune system, disease resistance and their capability of heat tolerance in indicine as compared to the taurine cattle. The differences in the target genes were further elaborated by Panther classification system [67] in order to predict the gene ontology of the potential target genes. The altered targets in indicine targeted binding (45%), biological regulation (11%), localization (8), response to stimulus (9%), signal transduction (6%), immune system (1%), biological adhesion (1%) etc. all categorized into biological process, cellular components and molecular function in (Fig 6A, 6B and 6C). A significant increase in the target genes can be observed in the indicine genome as compared to the taurine genome in (Fig 6D) representing the overall effect of the target genes in both sub-species. The increasing behavior of the target genes in various receptors, signaling, biosynthesis and stress pathways highlight the adaptation of indicine over the tropical and temperate habitat.

The potential seed mutations have a greater impact as enriched by the pathway classification, but individual miRNA, when studied for immunity and heat tolerance, showed a possible change in their targets. Bta-mir-2284e had a seed of AGUUCGU with 6 conserved targets (i.e. HNRNPU, HRB, LINGO1, RAB2A, TFAP2B, and NDST1) whereas, in indicine, a single mutation in the seed region AGUUCAU caused the loss of all these conserved targets. However, three genes associated with immunity were targeted by bind-mir-2284e. The seed region of mir-2403 tended to be UCGGGAA with a potential of 4 targets but the targets varied significantly with a single mutation UCAGGAA. The gene encoding methyl CpG binding protein 2 (MECP2) is targeted by both the species, is a common target gene. Whereas the mutation in indicine also tended to target 3 immunity and one heat tolerance gene. The bta-mir-2285n-6 showed 387 conserved targets in case of taurine, although, the mutation incurred in their seed region from AAAACCC to AAAAACT left with 137 common targets as well as 3 targeted genes related to immunity. The mir-2285o-5 showed 4 potential target genes whereas mutations were observed after SNPs in indicine miRNA from AACCCGA to GACCCGA, altered bta-mir-2285o-5 target genes to one immunity gene. On the other hand, 3 unusual SNPs in bta-mir-2285l in the seed region from AAACCCG to AATCCAA showed 56 potential targets with 12 common targets in both indicine and taurine cattle. Three potential immunity target genes were also predicted. The mutation of bta-mir-6522, in the seed region from CGGAATT to TGGAATT resulted in 268 conserved targets. The mutation in indicine resulted in no common targets, though it showed 5 potential immunity genes and 2 potential target genes associated with heat tolerance genes as mentioned in S5 Table.

**Fig 7:**
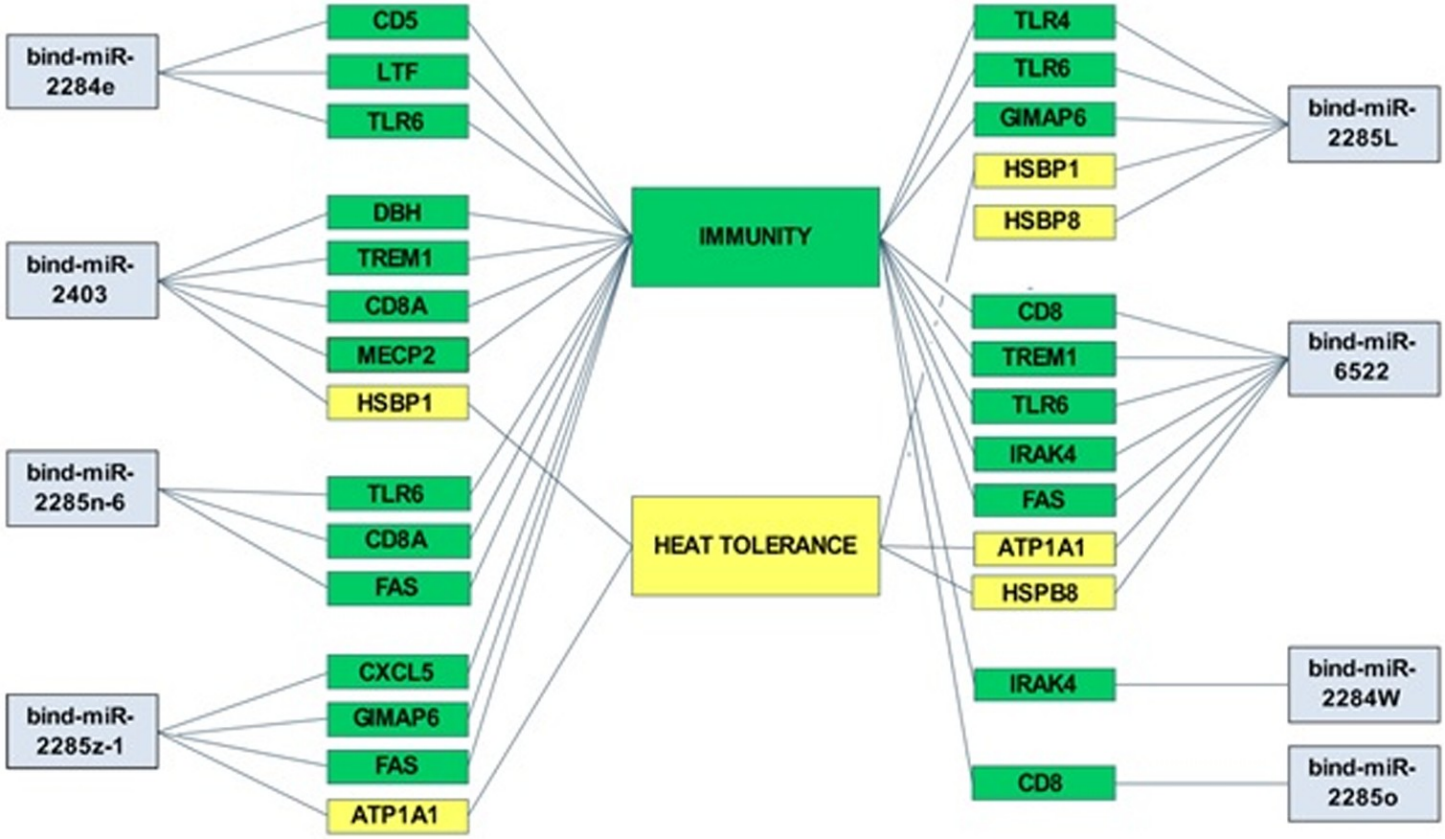
The association of potential miRNAs with heat and immunity related genes in *B*.*indicus* genome in network. The selected miRNAs showed seed mutation, potent for the alteration of the target gene regulation in the genome.

The SNP in the mir-2284w from AGAGTTT to AGAGTAT predicted 128 potential conserved target sites with an additional immunity gene in indicine. Similarly, bta-mir-2284z-1 showed 68 potential conserved target genes with 3 potential immunity and 1 heat tolerance genes in indicine as compared to taurine. (Fig 7 and S5 Table).

## Discussion

MiRNAs are known to play a crucial role in the regulation of the genes in animals as well as in plants. In the current study, we aligned all the known chordates particularly taurine miRNAs, to the indicine assembly, applying *de novo* and homology-based *in silico* searching. Furthermore, checking the secondary structures according to their MFEI at those loci for the extracted sequences, helped in identifying the conserved and new miRNAs in indicine genome.

Sahoo *et*.*al*. (2018) and Manku *et*.*al*. (2017) reported *in silico* miRNA identification approach as an accurate, precise, less expensive, fast and reliable method [37, 53]. The principle employed for the computational analysis include correct hairpins secondary structure, high degree evolutionary conservation between the closely related species and value of minimum folding energy [2, 5, 7, 9, 10]. We believe that the methodology opted here is sensitive enough to predict the miRNA homologs as performed by [5]. Moreover, Sengar *et*.*al*. (2018), Pande *et*.*al*. (2018) and Sengar *et*.*al*. (2018) experimentally validated a few of the targets of the miRNA genes discussed in our study recently, strengthening the reproducibility and credibility of the prediction criterion [6, 19, 60]. So, their behavior upon mutations can be deduced in the current study. The clustering of the miRNA across the genome sharing a single promoter helps to signify the accuracy of the target prediction of the regulatory genes as discussed by [68]. The findings support the microRNA target prediction in cattle and most extensive insight suggesting special functional roles in coordination, shedding the light on microRNA evolution and its variable function across large evolutionary distances.

Cui *et*.*al*. (2014) discussed the importance of miRNA structural stability in the post-transcriptional regulation [69, 70]. Although in animals, the miRNA binding is observed with non-perfect complementarity but the structural stability characterizes its ability to regulate target mRNA gene [14]. In parallel to Lukaszewicz *et*.*al*. *2015*, we also studied the *in silico* potential effect on precursor molecule of mir-2467, which revealed less stability of the structure of the miRNA on the basis of its minimum free energy index. The MFE-structure of taurine miRNA was found to have lower MFE value (-39.70) with a significant margin, as compared to indicine miRNA with an MFE of -29.80 with mutation of T/C at position 15 of 5’ arm of precursor mir-2467 [71].

We used panther ontology for explaining the biological process, molecular function and cellular components where we focused more on immunity parameters, disease resistance and heat tolerance for the target genes of the unique miRNAs. Interestingly, the increased number of targets resulted after the mutated miRNAs in indicine cattle revealed their potential in adaptive traits, MiRNAs, and innate immunity play a significant role in fine-tuning the gene expression [72, 73]. Many miRNAs have been studied and submitted to the miRBase regarding stress and metabolism [70, 73, 74]. MiRNAs tend to be the emerging novel regulators in heat-stress response [75, 76]. However, no study has yet been conducted for the miRNAs relating to heat and immune responses in Nellore cow. The current study conducted *in silico* genome-wide comparative study of miRNAs in indicine and taurine. We revalidated the role and general behavior of both sub-species from the published available data.

Olarte *et*.*at*. (2005) discussed the demerit of miRNA (mir-2305) down-regulation in the immunity of the taurine in brucellosis infection [77]. In the current study, we found the structural stability of mir-2305 in indicine cattle in comparison to taurine cattle to be associated with the enhanced response towards brucellosis in indicine. We propose that the mutation in the seed region might affect the stability of the miRNA and make it more stable resulting in decreased incidence of brucellosis in indicine.

The variable disease incidence in both subspecies has got the attention towards the tropical breeds which can provide the better insight towards the resistance and better tolerance [78]. In the present study, mir-2285 family (mir-2285l, 2285n-6) and mir-2284 family (2284-e, 2284z-1) were found to be associated with various TLRs thus regulating nonspecific, innate immune response by reducing pro-inflammatory responses [79, 80], suggesting that regulation of miRNAs play one of the key roles in indicine cattle tolerance to many inborn pathogens as compared to taurine cattle. Moreover, mir-2403, mir-2285n-6, mir-2285o-5 and mir-6522 have been shown to collaborate with CD8A (T-cell surface glycoprotein, CD8 alpha chain) that play a significant role in immune response development, T and B cell formation, inflammatory and proliferation responses [66]. This suggests that regulation of miRNAs might play one of the key roles in indicine cattle tolerance to many inborn pathogens. This may possibly hold some promising insight into adaptive, molecular and biological functional consequences in indicine cattle. In the present study, miRNAs have shown to regulate multistep TLR family, TREM1 [81], FAS [82], IRAK-4 [80], GIMAP and other immune receptors [83] for the appropriate response and are presented in the (Fig 7).

Silva *et*.*al*. (2013) enumerated the effect of heat stress as a major drawback in the development and reproductive life of livestock. Enhanced heat tolerance in indicine has been a characteristic feature as compared to taurine [22, 84]. The current study stands in parallel to Islam *et*.*al*. (2013) that mir-2285l and mir-6522 regulate HSPN8 (heat shock protein family B member 8) and mir-2403 and mir-2285l are potentially regulating HSBP1 (heat shock factor binding protein 1) [75, 76]. Furthermore, the oxidative stress and alteration of sodium and potassium pump are produced by heat stress which is critical for maintaining the homeostasis. There is a significant correlation between ATP1A1 and heat tolerance as described by [85]. Our study supports the association of mir-6522 and mir-2284z-1 with the regulation of ATP1A1 gene, which contribute an important role in the regulation of heat tolerance in indicine.

In the current study, we end up finding some interesting results besides the annotation of indicine miRNA. This is the first of a kind *in silico* study in indicine cattle that reflects some of the very important key regulations for disease resistance, tolerance, and immunity enhancements. The future perspective of the current research relies on the extended study and further experimentation which will augment the analysis and explore the significance of the regulatory networks in indicine genome.

## Supporting information

S1 Fig The workflow for the prediction, annotation, variant analysis and target identification of Bos indicus miRNAs. Precursor miRNAs of *B*. *taurus* taken from miRBase v.21 were mapped to the genome assembly of B. indicus. The missing precursors were searched by homology-based searches using their mature sequences and finally validated them for their secondary structures and energy before annotated as indicine miRNA.
(TIF)

S2 Fig Scheme adapted for the novel prediction of Bos indicus miRNA. The genome-wide analysis was carried out by splitting the indicine genome into short overlapping DNA segments. On the other hand, mature miRNA sequences of chordates were aligned with the indicine genome assembly with zero mismatches, for homology-based predictions and 150nt flanking regions were excised. The candidate precursors were filtered and redundancy was removed. Novel miRNAs were predicted on the basis of SVM probability (≥ 0.99), MFEI ≤-0.85 and secondary structure validation using MFold.
(TIF)

S1 Table Annotation of all the found miRNAs in indicine genome. (BED)

S2 Table Genome-wide d*enovo* prediction of mir-1264 from chordates as a data source using mirPara. (XLSX)

S3 Table Novel prediction of miRNA in indicine from chromosome Y. (XLSX)

S4 Table Polycistronic clustering of miRNA upstream with varying intergenic distances. (XLSX)

S5 Table Target Effect in addition to other putative target genes in Taurine and indicine. (XLSX)

S1 File Phylogenetic analysis of Novel predicted miRNAs by neighbor-joining using bootstrap 1000 replicates. (DOCX)
