# Supplementary figures and images for: In silico identification of conserved miRNAs and their selective target gene prediction in indicine (Bos indicus) cattle
Source: PLoS One. 2018 Oct 26;13(10):e0206154. doi: 10.1371/journal.pone.0206154 (PMC6203363; doi:10.1371/journal.pone.0206154)

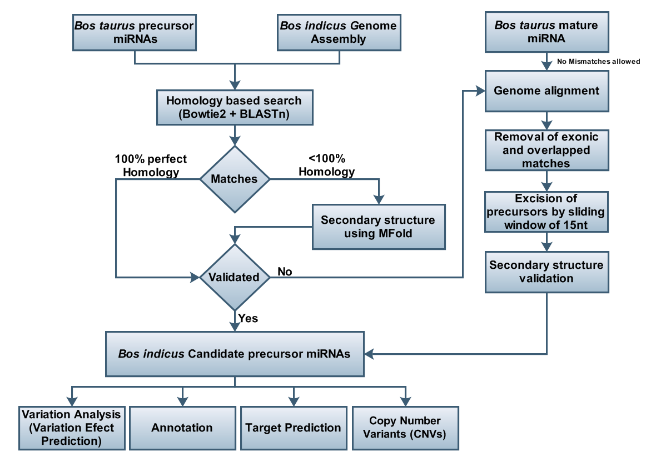

Supplement: S1 Fig — Precursor miRNAs of B. taurus taken from miRBase v.21 were mapped to the genome assembly of B. indicus. The missing precursors were searched by homology-based searches using their mature sequences and finally validated them for their secondary structures and energy before annotated as indicine miRNA. (TIF) [file pone.0206154.s001.tif]

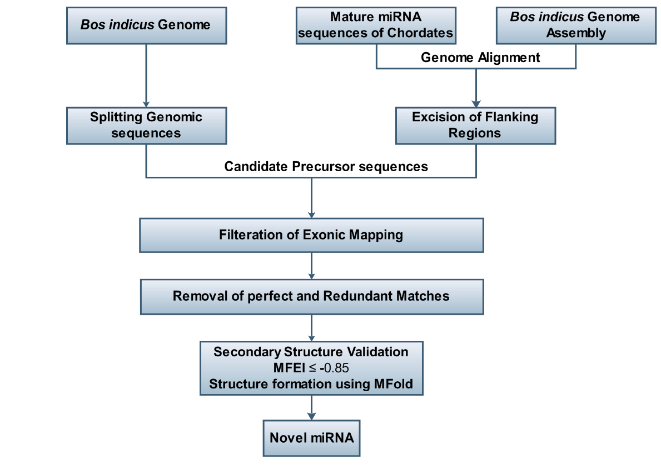

Supplement: S2 Fig — The genome-wide analysis was carried out by splitting the indicine genome into short overlapping DNA segments. On the other hand, mature miRNA sequences of chordates were aligned with the indicine genome assembly with zero mismatches, for homology-based predictions and 150nt flanking regions were excised. The candidate precursors were filtered and redundancy was removed. Novel miRNAs were predicted on the basis of SVM probability (≥ 0.99), MFEI ≤-0.85 and secondary structure validation using MFold. (TIF) [file pone.0206154.s002.tif]
