# Supplementary material for: In silico identification of conserved miRNAs and their selective target gene prediction in indicine (Bos indicus) cattle
Source: PLoS One. 2018 Oct 26;13(10):e0206154. doi: 10.1371/journal.pone.0206154 (PMC6203363; doi:10.1371/journal.pone.0206154)
Supplement: S1 File — (DOCX) [file pone.0206154.s008.docx]

Neighbour joining trees are based on p-distance method were constructed for the novel precursors together with its close homologous miRNAs that were BLAST searched from miRbase. Only bootstrap values higher than 30 were considered. All the trees were constructed within 1000 replicates. Evolutionary analyses were conducted in MEGA7. The predicted novel miRNA’s can evolve from very distant species, during the course of cattle evolution over 10,000 years ago. [1]

### Figure 1 The evolutionary history was inferred for bind-nov-1 using the Neighbour-Joining method. The optimal tree with the sum of branch length = 1.40625000 is shown. The predicted miRNA was found closest to [Medicago truncatula](http://www.mirbase.org/cgi-bin/mirna_summary.pl?org=mtr) and [Amborella trichopoda](http://www.mirbase.org/cgi-bin/mirna_summary.pl?org=atr) explaining the diversity in the evolution over the period of time.





Figure 2 The evolutionary history was inferred for bind-nov-2 using the Neighbor-Joining method. The optimal tree with the sum of branch length = 1.33653846 is shown. The percentage of replicate trees in which the associated taxa clustered together in the bootstrap test (1000 replicates) are shown next to the branches.





Figure 3 The evolutionary history was inferred for bind-nov-3 using the Neighbor-Joining method. The optimal tree with the sum of branch length = 2.73671875 is shown. The percentage of replicate trees in which the associated taxa clustered together in the bootstrap test (1000 replicates) are shown next to the branches. Here the miRNA has a close relationship with human miRNA, presenting a less divergence in terms of evolution.



Figure 4 The evolutionary history was inferred for bind-nov-4 using the Neighbour-Joining method. The optimal tree with the sum of branch length = 1.72697368 is shown. The percentage of replicate trees in which the associated taxa clustered together in the bootstrap test (1000 replicates) are shown next to the branches. The close relevance can be seen be the shared clad with [Rhesus lymphocryptovirus.](http://www.mirbase.org/cgi-bin/mirna_summary.pl?org=rlcv)





Figure 5 The evolutionary history was inferred using the Neighbor-Joining method. The optimal tree with the sum of branch length = 1.71634615 is shown. The percentage of replicate trees in which the associated taxa clustered together in the bootstrap test (1000 replicates) are shown next to the branches [2]. The clad is diversely shared by Heliconius Melpomene and Glycine max





Figure 6 The evolutionary history was inferred using the Neighbor-Joining method. The optimal tree with the sum of branch length = 1.71718750 is shown. The percentage of replicate trees in which the associated taxa clustered together in the bootstrap test (1000 replicates) are shown next to the branches. Here the predicted novel miRNA has some sharing with its closely related species which are highly diverse.



Figure 7 The evolutionary history was inferred for bind-nov-7 using the Neighbor-Joining method. The optimal tree with the sum of branch length = 1.95809659 is shown. The percentage of replicate trees in which the associated taxa clustered together in the bootstrap test (1000 replicates) are shown next to the branches. The predicted miRNA harbours the clad with [Hevea brasiliensis](http://www.mirbase.org/cgi-bin/mirna_summary.pl?org=hbr) and Pristionchus pacificus.





Figure 8 The evolutionary history was inferred for bind-nov-8 using the Neighbor-Joining method. The optimal tree with the sum of branch length = 2.66943359 is shown. The percentage of replicate trees in which the associated taxa clustered together in the bootstrap test (1000 replicates) are shown next to the branches. The predicted miRNA, shares it’s clad with Drosophila virilise, a diverse species’ miRNA evolution



Figure 9 The evolutionary history was inferred for bind-nov-9 using the Neighbor-Joining method. The optimal tree with the sum of branch length = 0.94375000 is shown. The percentage of replicate trees in which the associated taxa clustered together in the bootstrap test (1000 replicates) are shown next to the branches. This miRNA shares the clad with its close related species, however the evolutionary drift makes it distant with its homologies.



Figure 10 The evolutionary history was inferred for bind-nov-10 using the Neighbour-Joining method. The optimal tree with the sum of branch length = 1.92578125 is shown. The percentage of replicate trees in which the associated taxa clustered together in the bootstrap test (1000 replicates) are shown next to the branches. The homologues species tend to be quiet distant from the predicted miRNA due to evolutionary distances.



Figure 11 The evolutionary history was inferred for bind-nov-11 using the Neighbour-Joining method. The optimal tree with the sum of branch length = 0.50000000 is shown. The percentage of replicate trees in which the associated taxa clustered together in the bootstrap test (1000 replicates) are shown next to the branches. The predicted miRNA has relatively conserved nature and makes a clad with closely related species showing close genetic distance



Figure 12 The evolutionary history was inferred for bind-nov-12 using the Neighbour-Joining method. The optimal tree with the sum of branch length = 1.34801136 is shown. The percentage of replicate trees in which the associated taxa clustered together in the bootstrap test (1000 replicates) are shown next to the branches. The predicted miRNA has a close relevance with Drosophila melanogaster’s miRNA



**References:**

1. Pitt D, Sevane N, Nicolazzi EL, MacHugh DE, Park SD, Colli L, et al. Domestication of cattle: Two or three events? Evolutionary Applications. 2018.
